# Pathogenic Role of Adipose Tissue-Derived Mesenchymal Stem Cells in Obesity and Obesity-Related Inflammatory Diseases

**DOI:** 10.3390/cells12030348

**Published:** 2023-01-17

**Authors:** Julien Pestel, Ferdinand Blangero, Assia Eljaafari

**Affiliations:** 1 CarMeN Laboratory, Inserm U1060, University Claude Bernard Lyon 1, INRAE U1397, 69310 Pierre Bénite, France; 2 Research Department, Hospices Civils de Lyon, 69310 Pierre Bénite, France

**Keywords:** adipose tissue-derived mesenchymal stem cells, mesenchymal stem cells, obesity, plasticity, chronic inflammatory disease, cancer, Th17, IL-17 secreting cells, psoriasis, rheumatoid arthritis, multiple sclerosis, Crohn’s disease

## Abstract

Adipose tissue-derived mesenchymal stem cells (ASCs) are adult stem cells, endowed with self-renewal, multipotent capacities, and immunomodulatory properties, as mesenchymal stem cells (MSCs) from other origins. However, in a pathological context, ASCs like MSCs can exhibit pro-inflammatory properties and attract inflammatory immune cells at their neighborhood. Subsequently, this creates an inflammatory microenvironment leading to ASCs’ or MSCs’ dysfunctions. One such example is given by obesity where adipogenesis is impaired and insulin resistance is initiated. These opposite properties have led to the classification of MSCs into two categories defined as pro-inflammatory ASC1 or anti-inflammatory ASC2, in which plasticity depends on the micro-environmental stimuli. The aim of this review is to (i) highlight the pathogenic role of ASCs during obesity and obesity-related inflammatory diseases, such as rheumatoid arthritis, multiple sclerosis, psoriasis, inflammatory bowel disease, and cancer; and (ii) describe some of the mechanisms leading to ASCs dysfunctions. Thus, the role of soluble factors, adhesion molecules; TLRs, Th17, and Th22 cells; γδ T cells; and immune checkpoint overexpression will be addressed.

## 1. Introduction

Overweight and obesity are defined as excessive fat accumulation and can represent a danger for health. According to the World Health Organization (WHO), more than 1.9 billion adults were overweight in 2016, i.e., with a body mass index (BMI) ≥ 25, and among them 650 million were obese, i.e., with a BMI ≥ 30. Moreover, in the World Obesity Atlas 2022, WHO predicts that obesity prevalence will increase and should represent one billion people in 2030 [1]. In the WHO European Regional Obesity Report 2022, WHO reported that overweight and obesity affect almost 60% of adults including 23% obese individuals, and that overweight and obesity are prevalent not only among adults, but also among children, with nearly one in three children being overweight or obese (29% of boys and 27% of girls) [2]. Thus, obesity has been identified as a serious public health challenge globally and a major determinant of disability and death.

Indeed, obesity can induce severe complications, such as type 2 diabetes (T2D), hyperlipidemia, atherosclerosis, and cardiovascular (COV) diseases, and can aggravate other chronic inflammatory diseases and/or autoimmune diseases [3]. Moreover, obesity is also a poor prognostic factor for various types of cancer [4]. During obesity, adipose tissue (AT) is progressively infiltrated by inflammatory immune cells, which substitute to resident cells, such as M2 macrophages and regulatory T cells (Tregs) [5]. Thus, Nishimura demonstrated that the first immune cells, which infiltrate AT, are the CD8+ cytotoxic T lymphocytes with the goal to kill hypertrophic adipocytes. This infiltration is associated with a concomitant decrease in resident CD4+ Tregs and is followed by CD8+-T cell-dependent homing of type 1 macrophages (M1) eager to eliminate dead adipocytes. This results in the formation of crown-like structures, pathognomonic of AT inflammation, where adipocytes are encircled by CD8+ T cells and macrophages. Genetic depletion of CD8+ T cells in mice fed with a high fat diet also allowed researchers to demonstrate the implication of CD8+ T cells not only in obesity, but also in insulin-resistance development [6]. Along with CD8+ T cells, helper type 1 T lymphocytes (Th1) and other T lymphocyte subsets, such as natural killer T cells (NKT), mucosal T cells (MAIT), type 17 T helper cells (Th17) cells, and γ/δ T cells, have also demonstrated their implication in AT inflammation. Such an infiltration of inflammatory cells among AT results in the development of a pro-inflammatory environment, which decreases adipocyte insulin sensitivity and contributes to T2D pathogenesis [7]. In this context, we have previously described the leading role of obese adipose tissue-derived mesenchymal stem cells (ASCs) in the initiation of AT inflammation by demonstrating that obese, but not or much less, lean ASCs polarize T cells towards the Th17 cell subset, which secretes IL17A/F. ASC-mediated polarization of Th17 cells led to an ASC–Th17–Monocyte axis with production of interleukin-17 A/F (IL-17A/F) by Th17 cells, interleukin-6 (IL-6) by ASCs, and IL-1β by monocytes [8], as well as the propagation of inflammation by IL17A/F [9]. Moreover, almost half of the Th17 cells were double secreting IL-17 and IFNγ, which characterizes pathogenic Th17 cells [8]. Pathogenic Th17 cells are known to play an important role in the pathogenesis of several chronic inflammatory diseases, such as psoriasis, rheumatoid arthritis, multiple sclerosis, bowel diseases, or cancer, to which obesity is associated [3]. Thus, pathogenic Th17 cells may be one of the links between obesity and obesity-related inflammatory diseases. However, ASCs’ dysfunction may also play a role in in most of these diseases.

We will address in this review the mechanisms leading to ASC dysregulation during obesity and obesity-related inflammatory diseases.

## 2. Characteristics of Healthy Mesenchymal Stem Cells (MSCs)

### 2.1. MSCs Discovery

The first description of mesenchymal stem cells (MSCs) was performed in 1867 by the German pathologist Julius Cohnheim. He observed that cells with a fibroblast morphology are able to migrate from bone marrow towards the wound site and repair it through collagen fiber deposits [10]. Thereafter, while culturing guinea pig bone marrow cells, Friedenstein and colleagues found a population of nonhematopoietic cells with fibroblastic morphology that adhered to culture plastic and were able to rapidly expand [11]. These cells were further demonstrated to differentiate into osteoblasts, chondroblasts, adipocytes, or even myoblasts. Besides their multipotential capacity, Prockop demonstrated their ability to migrate and acquire the phenotype of the targeted tissue [12]. Accordingly, along with bone marrow, MSCs have been identified in almost all tissues and organs [13] and can be easily isolated from AT [14], dental tissue [15], skin [16], or perinatal tissue, i.e., umbilical cord blood, or Wharton’s Jelly [17].

### 2.2. MSCs Housing

Within tissues, MSCs are located in a particular environment, the niche. The term niche was first used by Schofield in 1978 [18]. It is a complex and dynamic environment with continuous interactions between stem cells and endothelial cells, allowing them to stay in a quiescent state and ensure their self-renewal. There are as many niches as there are sources of MSCs, but most niches share common components. Stromal cells help to maintain stemness of MSCs though cell–cell adhesion and gap and cadherin junctions. The extracellular matrix provides the niche structure in which blood vessels contribute to carry nutrients and molecular signals and allow for exchanges between the niche and others tissues [19]. Nevertheless, not all niches integrate all these components. For example, in AT, ASCs reside in close vicinity with perivascular cells surrounding blood vessels [20]. The major niche factor that modulates quiescence and stimulates ASCs is the growth factor like fibroblast growth factor 2 (FGF2), which plays a crucial role in ASCs’ self-renewal [21] and increases their multipotential capacity [22].

### 2.3. MSCs’ Tissue Repair Properties

Due to their ability to differentiate into various cell types and migrate into different tissues, MSCs are considered as a good source of cells for tissue regeneration, or repair. They are used in numerous clinical trials for the reconstruction of tissues including bone [23], cartilage [24], myocardium [25], and nervous system, notably in Parkinson’s disease [26] or liver [27,28]. Three major strategies are used at present: (i) cell therapy, which consists of injecting MSCs; (ii) tissue engineering, which combines MSCs, scaffolds, growth factors, cytokines, and chemokines; or (iii) injection of MSC-derived extra-vesicles, which have demonstrated their efficiency in tissue repair [29].

In addition to their differentiation and proliferative capacities, healthy MSCs display immunomodulatory properties.

### 2.4. MSCs’ Immunomodulatory Properties

#### 2.4.1. In Vitro Experiments

During mixed-lymphocyte reaction (MLR) experiments, T cells from peripheral blood mononuclear cells (PBMC) exert a proliferative or cytotoxic function due to the allo-reaction. However, the presence of MSCs has been shown to inhibit these two functions [30,31]. Supporting these results, MSCs have been shown to modulate the T helper 1/T helper 2 (Th1/Th2) ratio, inhibit T cell proliferation and cytokine secretion, and increase the number of regulatory Tregs during co-culture with T cells [32]. Our team has also demonstrated that MSCs are able to inhibit the commitment of dendritic cells into professional antigen-presenting cells through activation of the Notch pathway [33]. B cell proliferation, as well as antibody secretion have also been demonstrated to be inhibited by MSCs [34].

#### 2.4.2. In Vivo Studies

Supporting these in vitro studies, the immunomodulatory properties of MSCs have also been demonstrated in vivo, at first in a baboon skin transplantation model, where MSC injections were able to delay skin graft rejection [35]. The in vivo efficacy of MSCs to inhibit auto- or allo-reactions has further been confirmed in various experimental animal models, such as in systemic lupus erythematous disease, Crohn’s disease, colitis, arthritis, multiple sclerosis, and transplantation [36,37,38]. Our research team, using a model of induced type 1 diabetes in male NOD mice, has demonstrated the ability of MSCs to prevent the occurrence of the disease [39].

In humans, the first demonstration of MSCs’ beneficial therapeutic effects was reported by K. Leblanc, who cured a grade IV graft versus host disease (GVHD) in a child, following injection of allogeneic MSCs [40]. Since then, their use in therapeutic protocols for the treatment of GVHD [41] and other immune disorders, such as systemic lupus erythematosus inflammatory bowel disease, multiple sclerosis, rheumatoid arthritis, or even in acute respiratory distress syndrome or COVID-19, is constantly increasing, as seen below in the list of current clinical trials using MSCs: https://clinicaltrials.gov/ct2/results?cond=mesenchymal+stem+cells&term=mesenchymal+stem+cells&cntry=&state=&city=&dist=, accessed on 26 December 2022. Already published trials are shown in Table 1.

## 3. Mechanisms Involved in the Induction of MSCs’ Functions

### 3.1. Immunomodulatory Properties

Low levels of HLA-class II and co-stimulatory molecule expression are likely to contribute to MSC immunomodulatory properties [82]. However, secretion of soluble factors and cell–cell contacts are also likely to be involved.

#### 3.1.1. Soluble Factors

Prostaglandin E2 (PGE2) was proposed to contribute to the immunosuppressive function of MSCs, since its expression was upregulated during co-culture of MSCs with PBMC, and PGE2 inhibited IL-2 cytokine secretion and T cell proliferation with a concomitant increase of IL-10 production [83]. The indoleamine-pyrrole 2,3-dioxygenase (IDO) is likely to be one of the key immunomodulatory factors secreted by human MSCs [84]. Indeed, IDO is an enzyme preponderantly secreted by stimulated MSC in humans, which catalyzes tryptophan conversion into kynurenine. Thus, tryptophan is involved in the proliferation of immune cells [85], while kynurenine enhances regulatory T cell differentiation [86] and inhibits T cell and natural killer (NK) cell proliferation [87]. In mice, IDO secretion by MSC is quite low, but nitric oxide (NO), which can be secreted at high levels by MSCs following inducible NO synthase (iNOS) activation, contributes to MSC immunomodulatory function by suppressing T cell proliferation through the inhibition of signal transducer and activator of transcription (Stat)5 phosphorylation [88]. MSCs are also known to produce the hepatocyte growth factor (HGF), which induces a switch from Th1 to Th2 lymphocytes and induces IL-10 secretion by CD14+ monocytes via the activation of the Ras-dependent extracellular signal-regulated kinase (ERK)1/2 pathway [89]. Moreover, transforming growth factor-β (TGF-β) secretion by MSCs has been shown to suppress the allergic response in a mouse model of asthma, by recruiting Tregs towards the pulmonary site and decreasing eosinophil infiltration [90]. Finally, MSCs through secretion of IL1RA are also able to inhibit differentiation of B lymphocytes and promote polarization of macrophages towards the anti-inflammatory M2 subtype [91].

In addition to soluble factors, immunomodulation of MSCs also involves cell–cell contact.

#### 3.1.2. Cell–Cell Contact

##### Adhesion Molecules

Several studies have reported that cell–cell contact is required for MSCs immunomodulation. Thus, Intercellular Adhesion Molecule-1 (ICAM-1) and Vascular Cell Adhesion Molecule-1 (VCAM-1) were shown to play a critical role in MSCs’ immunosuppression, as assessed by their neutralization or genetical deletion. Indeed, co-culture of ICAM-1- and VCAM-1-deficient MSCs with T cells resulted in the restoration of T cell proliferation. Reciprocally, IFNγ, combined with TNFα or IL-1β, increased the expression of these adhesion molecules in MSCs, which resulted in the improvement of their immunomodulatory capacities, as assessed by marked inhibition of T cell proliferation [92].

##### Galectin-1

Galectin-1 has also been involved in the induction of MSCs’ immunomodulatory properties, since galectin-1 knockdown by RNA interference restored CD4+ T cell and CD8+ T cell proliferation, inhibited MSCs-mediated transition of CD4+ T cells from Th1 to Th2, and therefore increased the levels of Th1 cytokine production, notably interferon-γ (IFN-γ) and tumor necrosis factor-α (TNFα) [93]. Cell–cell contacts were not analyzed in this study but may account for most of galectin-1 immunomodulatory effects, since cell surface galectin-1 has been shown to be much more efficient in killing T cells than soluble galectin-1 [94].

#### 3.1.3. Immune Check Point Expression

Immune check point (ICP) overexpression is another way used by ASCs to inhibit immune responses. Indeed, those molecules are able to induce exhaustion of T and NK cells following binding to relevant receptors [95]. Such a mechanism is largely used by cancer- and virus-infected cells [96]. However, we demonstrated that ICP expression is also increased in AT during obesity and is promoted in vitro by ASCs following interaction with immune cells [97], suggesting T cell exhaustion as an additional immunomodulatory mechanism by which ASCs inhibit T cell functions.

### 3.2. Pro-Inflammatory Properties

**Figure 1:**
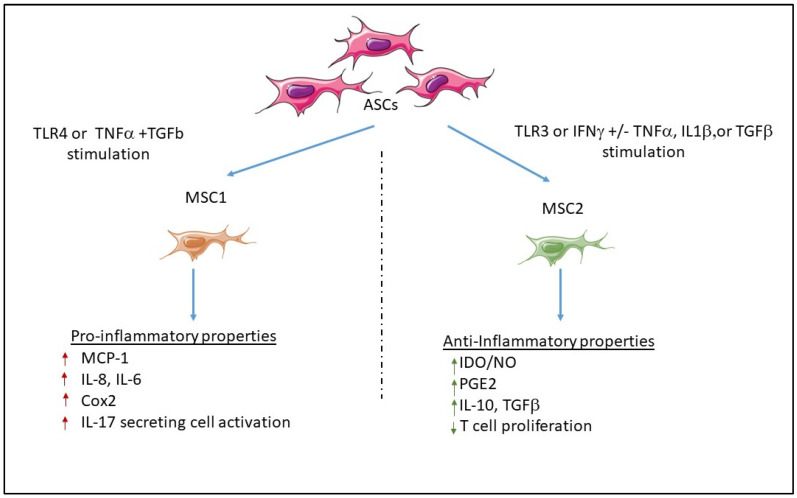
Classification of ASCs according to two main classes. Following stimulation, MSCs can display opposite properties and become pro-inflammatory MSC1 or anti-inflammatory MSC2.

MSCs have thus demonstrated a particular interest in cell therapy, tissue engineering, and/or immune modulation. However, recent findings indicate that MSCs can also display pro-inflammatory properties depending on paracrine stimuli, as shown in Figure 1.

#### 3.2.1. Mechanisms Involved in the Promotion of Anti- or Pro-Inflammatory MSCs

##### Inflammatory or Anti-Inflammatory Cytokine Effects

The first molecule that was reported to induce immunomodulation in co-culture experiments of MSC with lymphocytes was IFNγ. Indeed, IFNγ was shown to inhibit T cell proliferation in human cells, through activation of indoleamine 2,3-dioxygenase (IDO) in MSC [98]. Then, Ren et al. demonstrated that IFNγ combined with TNFα, IL-1α, or IL-1β activated iNOS in mouse MSCs, which resulted in NO increase and subsequent immunomodulation, as shown in a GVHD mouse model [99]. The particular role of IFNγ was thereafter confirmed using experiments in which mouse recipients of IFNγ(-/-) T cells did not respond to MSC treatment and succumbed to GVHD. Reciprocally, administration of IFNγ-pre-treated MSCs improved GVHD [100]. Another way to drive the fate of MSCs towards the anti-inflammatory or pro-inflammatory state is related to the action of TGFβ, combined with IFNγ or TNFα. Indeed, when combined with IFNγ, TGFβ favored MSC immunomodulation, as demonstrated by (i) increased promotion of Tregs differentiation, (ii) inhibition of T cell proliferation, and (iii) increased IDO production [101]. However, when combined with TNFα, TGFβ rather induced overexpression of monocyte chemoattractant protein (MCP)-1, IL-8, and cyclo-oxygenase-2 (COX)-2 in MSCs, which then initiated inflammation [102].

##### Role of Toll-Like Receptors

Waterman et al. proposed that two phenotypes of pro-inflammatory or immunosuppressive MSCs, named MSC 1 or MSC2, respectively, could be differentially activated through toll-like receptor (TLR) 4 or 3, respectively. Indeed, they found that LPS was able to activate MSC1 through TLR4, a pattern recognition receptor that recognizes conserved pathogen-associated molecular patterns (PAMPs) from bacterial origin, while polyinosinic-polycytidylic acid (Poly:IC) activated MSC2 through TLR3, which recognizes PAMPs from viral origin. Thus, LPS-stimulated MSC1 expressed higher levels of IL-6 and IL-8 mRNAs than poly(I:C)-stimulated MSC2, which inversely expressed immunomodulatory factors, such as IDO, PGE2, and IL-10, at much higher levels than LPS-stimulated MSC1 [103]. Similar observations have been made for ASCs, which, depending on TLR3 or TLR4 stimuli, could behave as immunomodulatory or inflammatory cells, respectively [104].

Thus, ASC plasticity may contribute to the pathogenesis of obesity and obesity-related inflammatory diseases.

## 4. Contribution of ASCs in Obesity and Obesity-Related Inflammatory Diseases

### 4.1. Obesity

During obesity, the number of adipocytes increases (hyperplasia) as well as their size (hypertrophy). Indeed, during obesity, compression of the blood vessels by hypertrophic adipocytes reduces capillary density in AT. Even though the presence of larger vessels is observed, the blood flow to white adipose tissue does not increase. Thus, the supply of O_2_ to AT is restricted in obesity, as this has been demonstrated using hypoxyprobes [105]. Hypoxia activates hypoxia-inducible factor (HIF)1-α transcription in adipocytes and ASCs, which results in increasing the expression of fibroblast growth factor (FGF)-2 and vascular endothelial growth factor (VEGF)-A as well as metalloproteinases (MMP)-2 and 9, leading to increased ASCs proliferation and hyperplasia and AT remodeling [106]. Accordingly, HIF-1α mRNA expression in progenitor cells has been positively correlated with BMI and fat mass enlargement with increased proliferation of progenitor cells in obese individuals [107].

#### 4.1.1. Infiltration of Inflammatory Cells

The secretion of HIF1-α also results in increasing the levels of ASCs chemokine secretion, such as MCP-1 and CCL2, which attract inflammatory leukocytes from blood, as mentioned above, in the Introduction Section. Moreover, γ/δ T cells, which are resident in AT, increase in number due to increased proliferation and homing from blood mediated by high CCL2 and IL-6 levels. γ/δ T cells, which represent a bridge between innate and adaptive T cell responses, produce high levels of IL-17A in obese AT and contribute to AT inflammation and insulin resistance, as demonstrated by the reduction of these two pathological processes in mice deficient in TCRγ/δ and fed with a high fat diet [108]. Another bridge between innate and adaptive immunity is represented by MAIT innate-like T cells, which express a semi-invariant TCR-α chain and are restricted by MR1, a CMH-related molecule presenting bacteria or yeast antigens. During obesity, MAIT cell frequency decreases in blood but increases in visceral AT, due to their attraction by CCL20, a chemokine secreted by mature adipocytes in correlation with increased BMI [109], but also by adipocyte precursors under the govern of IL-1β and IL-17 [110]. In addition to Th17 cells and γ/δ T cells, MAIT cells also produce IL-17 and have been demonstrated to promote inflammation within AT and intestine leading to insulin resistance and impaired glucose and lipid metabolism, with the help of a MR1-/- mouse model [111].

#### 4.1.2. Modification of the Secretome Profile

In such an AT environment resulting from infiltration of inflammatory immune cells, the secretome profile of ASCs moves towards a pro-inflammatory pattern secreting IL-1β, IL-6, and IL-8 cytokines with activation of the NLRP3 (NOD-, LRR-, and pyrin domain-containing protein 3) inflammasome [112] and of nuclear factor κB kinase (NFkB), c-Jun-N-terminal kinase (JNK), and mitogen-activated protein kinase (MAPK), known to inhibit insulin signaling through inhibition of the insulin receptor substrate (IRS) tyrosine phosphorylation [113]. Increased secretion of adipokines involved in weight gain, lipolysis, and insulin-resistance, such as leptin and resistin, was also observed in this altered secretome profile, concomitant with a decrease in adipokines favoring immune suppression, insulin sensitivity, and weight loss, such as IL-10, TGFβ, and adiponectin [114,115].

#### 4.1.3. Alteration of ASC Properties

This inflammatory environment is shown to compromise the immunomodulatory capacities of ASCs from obese and T2D patients, with a lower ability to inhibit T and B cell proliferation and increased HLA-class II molecule expression [112]. Obese ASCs, and more particularly visceral ASCs, have also been reported to decrease their adipogenic potential in comparison to lean ASCs, due to a lower expression of adipogenic factors, notably PPARγ, and FABP4 [116]. They also lose their multipotent properties, with a decrease in stemness gene expression, such as HOXC10 and TBX15, which are involved in embryonic development, and ACTA2 involved in multilineage differentiation [117]. Due to activation of MMPs secretion by ASCs and alterations of extracellular matrix protein production, obese ASCs are also involved in the remodeling of AT leading to increased fibrosis [118]. Thus, the donor metabolic profile is likely to compromise the functions of ASCs in modulating immune responses or repairing tissues.

#### 4.1.4. Metaflammation

**Figure 2:**
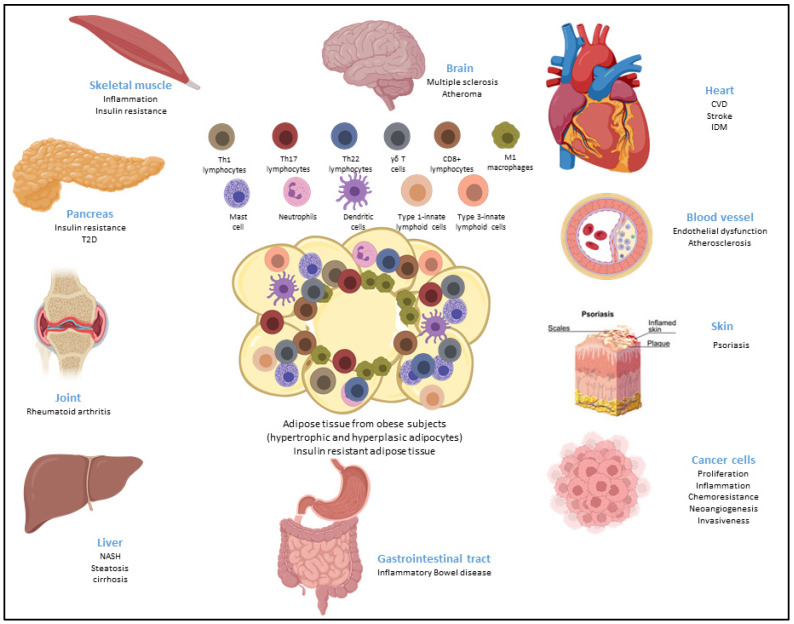
Metaflammation is characterized by chronic low-grade inflammation induced by metabolic alterations, such as during obesity, and the propagation of inflammation to other metabolic tissues or organs, thus aggravating the prognostic of various inflammatory and autoimmune diseases.

Obese ASCs were also shown to polarize immune cells towards an inflammatory profile, as reported for inflammatory macrophages [119] and as mentioned above for Th17 cells [8]. Since those cells secrete cytokines, such as IL-1β, TNFα, IL-6, IL17, and IFNγ, that are able to spread inflammation towards ASCs and other environmental cells, such as adipocytes, fibroblasts, and endothelial cells, a vicious circle is initiated, leading to the development of a low-grade inflammation within AT and insulin resistance, which spreads towards other metabolic tissues and organs, resulting in metaflammation that is characterized by a chronic low-grade inflammation state induced by metabolic alterations [120]. Thus, due to the presence of metaflammation, obesity aggravates the prognostic of multiple pathologies, notably cancer and chronic inflammatory and autoimmune diseases, as described below and as shown in Figure 2.

### 4.2. Cancer

Obesity is one of the strongest risk factors of cancer due to decrease of overall survival and resistance to anti-cancer therapy, such as in pancreatic, gastric, lung, ovarian, breast, prostatic, gastro-intestinal, and blood cancers [121,122,123,124,125].

#### 4.2.1. Role of Obese ASCs in Increased Vascularization and Tumor Growth

Among other actors, ASCs are involved in cancer progression. Indeed, ASC transplanted in a murine model of cancer showed capacities to (i) migrate from WAT towards tumors, (ii) populate the tumor microenvironment inside perivascular niches in which they are incorporated as pericytes, and (iii) differentiate into adipocytes to provide a source of energy. Thus, ASC recruitment is associated with enhanced vascularization and tumor growth and is greatly increased in obesity, as assessed by a six fold increase of ASC frequency in the systemic circulation of tumor-bearing obese mice compared to lean mice [126]. Hyperplasia may account for this increase as well as homing, which preponderantly depends on the SDF-1/CXCR4 axis with increased expression of CXCR4 in cancer cells and SDF-1 secretion by ASCs. IL-8 and CXCL-1, whose secretions are increased in obese ASCs, have also been shown to contribute to the recruitment of ASCs into the tumor site [127,128,129,130].

#### 4.2.2. Differentiation of Obese ASCs into Carcinoma-Associated Fibroblasts

Another strategy used by ASCs to promote tumor progression is their ability to differentiate into carcinoma-associated fibroblasts (CAFs) under the influence of cancer-derived factors. Using conditioned medium from breast cancer cells or exosomes from ovarian cancer cells, two studies have demonstrated that ASCs are able to differentiate into fibroblasts through increased secretion of TGF- β1 by cancer cells and subsequent activation of the Smad-3 signaling pathway [131,132]. CAFs display a myofibroblast-like morphology and increased invasiveness capacities, due to increased tenascin C and α-smooth muscle actin (SMA) expression [131]. They also promote tumor growth and angiogenesis through increased SDF1 and CCL5 secretion. Interestingly, Strong et al. demonstrated a higher conversion of obese ASCs into CAFs upon co-culture with cancer cells compared to lean ASCs [133].

#### 4.2.3. Role of the Obese ASC Secretome

ASCs’ secretome modifications in obesity result in increased production of growth factors, cytokines, chemokines, and adipokines. Among them, angiogenic factors, such as VEGF-A and PDGF; cytokines, such as transforming growth factor-β1 (TGF- β1), insulin-like growth factor (IGF), IL-6, and IL-8; and chemokines, such as C-X-C Motif Chemokine Ligand (CXCL)1/2/5 and C-C motif chemokine ligand (CCL)-2, also named monocyte chemoattractant protein 1 (MCP-1), have been shown to play important roles in stimulating cancer progression [130]. Increased secretion of hormones, such as leptin, adipsin, and survivin, by obese ASCs is also involved in cancer development.

Indeed, leptin, which is an adipokine secreted by mature adipocytes but also by ASCs from obese individuals, is considered as a pro-tumoral factor in various cancer types through (i) activation of the AKT pathway, which induces increased levels of fatty acid synthase (FAS) and HSP90 [134]; (ii) activation of MAP kinases and PKA, resulting in increased activation of NO synthase and COX-2 [135] and leading to increased vasculogenesis and tumor growth [136,137]; or (iii) activation of the Janus kinase (JAK)/STAT3 signaling pathway, promoting by this way cancer cell proliferation, migration, and invasion [138]. Leptin’s role in ASC-mediated tumor progression was demonstrated by the use of leptin silencing shRNA in obese ASCs, which prevented the enhanced proliferative effects of obese ASC on breast cancer cells following co-culture of ASCs with breast cancer cells [139].

Adipsin, the levels of which are correlated to body weight and leptin levels, is secreted by ASCs within AT. It is a target of PPARγ and is involved in the regulation of the C3 complement. Adipsin does not alter lipid or glucose metabolism in obesity, but rather protects pancreatic β-cells from failure, dedifferentiation, or death [140,141]. Nevertheless, its role in enhancing human breast cancer growth was demonstrated using adipsin knockout (KO) mice with breast cancer [142].

Survivin is secreted by ASCs, especially obese ASCs. This adipokine activates tumor-associated macrophages and inhibits apoptosis, thus favoring cancer progression [143].

#### 4.2.4. Role of Obese ASCs in the Attraction and Polarization of Pathogenic IL-17 and IL-22 Secreting Cells

Attraction/and or polarization of IL-17 secreting cells is another way by which ASCs may mediate tumor progression in obesity. Indeed, IL-17A/F secreted by pathogenic Th17 cells has been shown to contribute to growth and metastasis of numerous cancers [144]. Thus, IL-17 stimulates the production of CXCL1/CXCL5/Granulocyte Colony-Stimulating Factor (G-CSF) by ASCs and other cells present in the tumor microenvironment. This leads to (i) the recruitment of myeloid cells and MDSCs, which promote angiogenesis and suppress tumor immunity; and (ii) increased paracrine IL-6 production, known to increase tumor growth and survival [128].

γ/δ T cells may also be involved, as they are associated with a poor prognosis in obese individuals, due to their high levels of IL-17 secretion, whereas they are considered as a positive prognostic factor in cancer-bearing lean patients due to their ability to secrete Granzyme B and IFNγ [145].

Th22 cells, which secrete IL-22, are recruited within AT of obese individuals together with Th17 cells, and CD4+ cells double secreting IL-17 and IL-22 [146]. IL-22 plays a particular role in tumorigenesis since it contributes to ASCs’ homing towards tumoral sites through induction of CXCL1 secretion by tumoral cells expressing IL22R. Thus, IL-22 levels have been found to be markedly elevated in blood of tumor-bearing obese mice [147].

#### 4.2.5. Role of Obese ASCs in Epithelial Mesenchymal Transition of Cancer Cells

The epithelial mesenchymal cell transition (EMT) converses cancer cells into cells with stemness properties, such as self-renewing and increased ability to invade and migrate due to loss of E-cadherin, but increased N-cadherin, fibronectin, and/or vimentin expression. It is often activated during cancer invasion and metastasis, and allows for the autocrine proliferation and dissemination of metastatic cells [148]. EMT have been shown to increase EMT induction through SDF1 secretion and binding to CXCR4 and CXCR7 in cancer cells [149] Moreover, EMT was reported to increase in tumor-bearing obese as compared to lean mice and decrease following injection of D-CAN, a killer peptide targeting ASCs [150].

**Figure 3:**
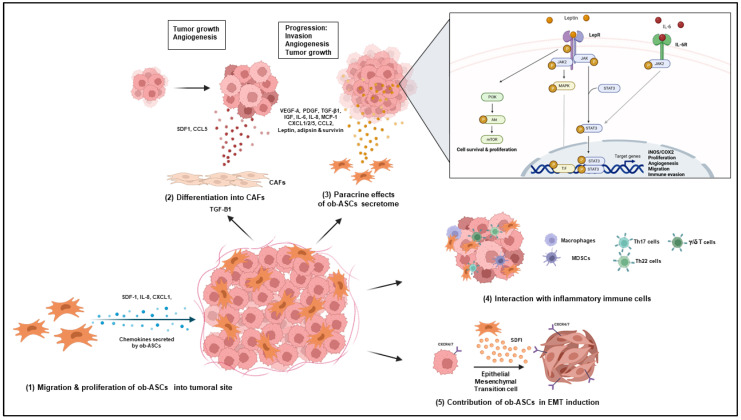
Impact of ob-ASCs on cancer progression. Ob-ASCs are able to migrate towards the tumor site, differentiate into carcinoma-associated fibroblasts (CAFs), and secrete pro-tumorogenic soluble factors. This may result in cancer progression with increased tumor growth, neo-angiogenesis, invasiveness, and/or epithelial mesenchymal transition (EMT).

Taken together, these data demonstrate the implication of ASCs/MSCs and particularly obese ASCs in cancer where they promote growth, invasion, metastasis, and chemoresistance, as summarized in Figure 3.

### 4.3. Chronic Inflammatory and Autoimmune Diseases

#### 4.3.1. Multiple Sclerosis

##### Role of IL-17

Multiple sclerosis (MS) is an autoimmune demyelinating and neurodegenerative disease of the central nervous system with increased levels of pro-inflammatory cytokines, such as TNFα, IFNγ, and IL-17A in periventricular foci and blood. IL-17 was demonstrated to play an important role in an experimental murine model of autoimmune encephalomyelitis (EAE), which mimics MS. Indeed, the use of IL-17-/- mice demonstrated delayed onset of EAE and reduced severity scores [151]. Moreover, treatment with anti-IL-17-neutralizing Abs resulted in partial protection from EAE [152]. Other factors, such as IL-6, TNFα, and IFNγ, also contributed to the disease [151,152]. Moreover, not only pathogenic Th17 cells secreting IL-17 plus IFNγ, GM-CSF, and/or TNFα are likely to contribute to the development of the disease, but also γ/δ T cells activated by IL-1β and IL-23 independently of the antigen. Indeed, γ/δ T cells participate in the recruitment of IL-1β-producing neutrophils and monocytes, which prime pathogenic T cells. Thus, this results to an inflammatory positive feedback loop [153].

##### Association with Obesity

Early childhood and adolescent obesity is a risk factor for MS, essentially in females, as demonstrated by epidemiological studies who found a twofold increase in the obese populations at age 20, on average [154]. Moreover, obesity is linked to a weaker response to multiple sclerosis treatments [155]. The underlying mechanisms linking obesity to EAE are likely to be chronic inflammation, since increased serum levels of adipokines, such as leptin and resistin, are associated with autoimmune diseases, including MS [156]. Moreover, increased production of IL-17 in AT and blood of obese individuals may contribute to EAE development, since IL-17A has been unequivocally found to be pathogenic in EAE, as mentioned above [153]. Another hypothesis concerns the defect in vitamin D, which often occurs in obese individuals [157,158], since lower levels of vitamin D have been associated with increased risks of MS and severe disease progression [159].

##### Contribution of ASCs and MSCs

MSCs isolated from patients with MS display reduced ex vivo clonogenic potential, premature senescence, and accelerated shortening of telomere terminal restriction fragments [160]. In addition, they display reduced neuroprotective potential due to the dysregulation of their antioxidant responses. Indeed, reduced secretion of superoxide dismutase1 (SOD1) and gluthatione S transferase (GST) by MSC were negatively correlated to the duration of the progressive phase of MS [161].

The role of ASCs in EAE has been indirectly proven by their beneficial therapeutic effects, since administration of healthy ASCs has been shown to delay the disease onset and reduce disease severity by inhibiting immune cell proliferation [162]. Supporting these results, Yousefi et al. demonstrated that transplantation of ASC-overexpressing leukemia inhibitory factor (LIF), a neurotropic agent, and IFNβ, an anti-inflammatory cytokine, promoted the recovery from EAE due to (i) increased immunomodulatory effects, (ii) reduction of the extent of demyelination, (iii) enhancement of the number of oligodendrocytes, and (iv) increase of the amount of MBP protein and further myelin production [163]. However, the anthropometric status of ASC donors appears of particular importance, since transplantation of ASCs from obese donors exacerbated EAE disease by increasing the expression of pro-inflammatory cytokines and inducing proliferation and differentiation of CD4 and CD8+ T cells. This resulted in enhanced demyelination of the central nervous system and enhanced immune cell infiltration into the central nervous system compared to lean ASCs [162]. Thus, as compared to lean ASCs, Ob-ASCs did not inhibit, but rather exacerbated the disease. This could be related to the increased secretion of inflammatory cytokines, such as IL-1b, which is known to activate expansion of antigen-driven T cell expansion and differentiation [164] or activation of pathogenic Th17 cells, secreting both IL-17 and IFNγ, two cytokines involved in EAE pathogenesis, as demonstrated by us in vitro [8].

#### 4.3.2. Psoriasis

Psoriasis is a chronic inflammatory disease of the skin where keratinocytes hyperproliferate due to the interplay between genetic components, immune dysfunction, and environmental factors.

##### Role of Inflammatory Mediators

Psoriasis is characterized by proliferation of Th1, Th17, and Th22 cells that produce IFNγ, IL-2, IL-17A/F, and IL-22 and induce TNFα and IL-6 secretion by surrounding cells [165]. Leptin and resistin are found at higher levels in psoriasis patients. Leptin’s ability to promote increased secretion of pro-inflammatory cytokines by keratinocytes may explain the correlation found between leptin plasma levels and psoriasis severity [166,167]. Therefore, increased secretion of pro-inflammatory adipokines may account for one of the links between psoriasis and obesity [168].

##### Association with Obesity

Psoriasis is associated with numerous comorbidities, such as psoriatic arthritis, cardiovascular disease, metabolic syndrome, and obesity [169]. Obesity is an independent risk factor for psoriasis incidence, since the prevalence of psoriasis is increased in obese people, and a graded positive association between BMI and relative risk of psoriasis is established [170]. Furthermore, obesity is more particularly associated with a higher risk of severe psoriasis (OR 2.23), as compared to mild disease (OR 1.46), thus supporting that obesity may aggravate existing psoriasis [171]. Moreover, obesity in childhood or adolescence is likely to precede severe psoriasis in adulthood [172]. Finally, leptin is likely implicated in the increased rates of psoriasis in obese individuals [171,173].

##### Role of MSCs

Angiogenesis and lymphangiogenesis are likely to participate to the pathogenesis of psoriasis, in which VEGF-A and VEGF receptors are highly expressed, even in the non-involved skin [174]. MSCs from the skin of psoriasis patients are reported to overexpress the proangiogenic factor VEGF and iNOS, as compared to MSCs from healthy donors, thus suggesting their implication in increased angiogenesis and inflammation [175]. Psoriatic MSCs also display lower immunomodulatory properties, as they are less able to inhibit T cell proliferation [176] In addition, the cytokinic expression profile of MSCs isolated from the skin of psoriatic patients demonstrated higher levels of and Th1- and Th17-type cytokines, but similar levels of M2- and Th2-type cytokines as compared to healthy MSCs, thus demonstrating an imbalance between the Th1/Th17 and Th2 cytokine expression profile. Thus, the hallmarks of psoriasis, such as the imbalance between Th1/Th17 and Th2 cytokines and the upregulated expression of VEGF and iNOS, were all detectable in MSCs, suggesting their contribution in psoriasis pathogenesis [177].

Finally, if beneficial, the effects of healthy ASC transplantation in this disease should prove the role of ASCs. However, clinical trials, in which safety has been demonstrated, are still in progress [178,179].

#### 4.3.3. Rheumatoid Arthritis

Rheumatoid arthritis (RA) is a chronic inflammatory disease targeting the synovium. During RA, synovial fibroblasts, also named syniviocytes, proliferate and attract blood leukocytes towards the synovial membrane. The levels of pro-inflammatory cytokines and MMPs strongly increase leading to cartilage and bone erosion.

##### Association with Obesity

Obesity is a controversial risk factor for RA, as assessed by epidemiological studies [180,181]. However, Crownson et al. reported that obesity may contribute to the rise of RA incidence, since obesity could explain 52% of increased incidence [182]. Moreover, their retrospective data analysis suggested that obesity precedes RA. Among others, a mechanism involved in obesity-mediated increased risks of RA may be low-grade inflammation of articular AT, resulting in increased adipokine and cytokine secretion by ASC and activation of Th17 cells.

##### Role of Th17 and MSCs/ASCs

Th17 cells play a central role in RA damages following infiltration in joints [183]. Thus, they have been shown by us to activate the secretion of IL-6, IL-8, and IL-1β by synoviocytes and to be in turn activated by MSCs and synoviocytes [184]. Moreover, IL-17 was shown by others to increase MMP13 gene expression by chondrocytes, which contributes to cartilage and bone erosion [185]. Interestingly, weak immunomodulatory properties of ASCs from RA patients, but increased ability to induce IL-17A secretion by T cells, led to the conclusion that the dysfunction of ASC may contribute to RA pathogenesis [186].

#### 4.3.4. Crohn’s Disease (CD)

Inflammatory bowel disease (IBD), with its two subtypes of Crohn’s disease (CD) and ulcerative colitis (UC), is a chronic inflammatory disorder of the gastrointestinal tract. CD is characterized by chronic inflammation and ulceration of the large and small intestine, with hyperplasia of the mesenteric fat adjacent to the inflamed region [187]. Mesenteric fat may contribute to the inflammatory response; it is produced by mesenteric adipocytes and is an important source of C-reactive protein in CD [188].

##### Association with Obesity

In CD, obesity has been inconsistently associated with increased prevalence of CD. Thus, some authors have reported that there is evidence of increasing degrees of obesity associated with increased risk of CD [189,190]. However, other studies indicate that obesity is not associated with CD incidence [191,192]. To reconcile these discordant studies, Blain et al. reported that obese patients were more prone to develop an active disease and to require hospitalization, but without any alteration of the long-term course of the disease. [193].

##### Role of ASCs

ASCs from mesenteric or subcutaneous AT show an inflammatory, proliferative, and invasive pattern, with defects in adipogenic capacities and immunomodulatory properties as compared to healthy ASCs. Moreover ASCs from CD patients exhibit high bacterial phagocytic and migratory capacities and HLA class II expression, suggesting their implication in inflammation-mediated intestinal tissue damages [194].

## 5. Conclusions

### 5.1. Lean Versus Obese ASCs/MSCs

In physiological conditions, MSCs and ASCs display the ability to differentiate into multiple cell types, exert immunosuppressive capacities, and are able to migrate towards wound sites. However, in a pathological context, MSCs display opposite properties. Thus, as compared with lean ASCs, ob-ASCs display decreased (i) differentiating capacities, (ii) immunomodulatory activities, (iii) stemness, (iv) self-renewal capacities, and (v) telomerase activity and length; however, they display increased ability to (vi) secrete inflammatory factors; (vii) activate inflammatory cells in their environment, such as macrophages, microglial cells, or T cells; (viii) induce pro-angiogenesis, tumor growth, invasiveness, EMT, and resistance to chemotherapy or radiation; (ix) or induce metabolic dysfunctions [115,116,117,195,196,197,198,199,200,201,202], as summarized in the graphical abstract. At the therapeutical level, when compared to lean ASCs/MSCs, ob-ASCs/MSCs have demonstrated an ability to aggravate instead of cure or prevent the disease once transplanted. Such an example has been reported in EAE, as described above [162], but also in another study performed in a mouse model where lean versus obese ASC were transplanted to repair renal artery stenosis. Authors reported an induction of inflammation and mitochondrial dysfunction in renal cells co-cultured with obese ASCs and a failure in decreasing serum creatinine and blood pressure in vivo, as opposed with the beneficial effects obtained with lean ASCs [203].

Thus, a better comprehension of the mechanisms leading to ASC/MSC dysregulation should help to prevent their dysfunctions. As an example, Ritter et al. demonstrated defective primary cilia in obese ASCs leading to alteration of differentiation and motility, which was rescuable by the low-dose inhibition of Aurora A or Erk1/2 [204]. In another report, inhibition of leptin secretion by a neutralizing antibody demonstrated its efficacy in reducing ob-ASC-mediated cancer proliferation [205]. Another example is given by our demonstration of the important role of ob-ASC interaction with immune cells in the development of inflammation among AT. This led us to block this interaction using omega-3 poly-unsaturated fatty acids and demonstrate the ability to block Th17 cell activation in this way [206]. New targets of cell–cell interactions are now under investigation.

### 5.2. Cell Therapy Programs

Due to their beneficial properties, healthy MSCs/ASCs present a great interest in the treatment of chronic and autoimmune diseases, in addition to regenerative medicine. They have also demonstrated an ability to improve metabolic disturbances and suppress body weight increase in a mouse model [207,208]. However, particular attention should be paid to the recruitment of ASC/MSC donors in cell therapy programs to avoid obese donors, as mentioned in reports willing to optimize MSC manufacturing processes, and to select optimal allogeneic donors [209,210].

## Figures and Tables

**Table 1:** MSCs’ use in human clinical trials.

General Indication	Clinical Indication	Cell Source	Injection	Phase	Results (Safety/Efficacy)	Trial Number	Ref
**Autoimmune** **disease**	Type 1 Diabetes Rheumatoid Arthritis Systemic Lupus	Auto BM Allo AT Allo UC Auto BM Allo AT Auto UC Allo UC	Systemic Systemic Systemic Local Local Systemic Systemic	N.A N.A N.A 2/3 1/2 1 1/2	Safe and clinical efficacy Safe and clinical efficacy Safe and clinical efficacy Safe and clinical efficacy Safe and trend for efficacy Safe and clinical efficacy Safe and clinical efficacy	NCT01068951 NCT03920397 **X** NCT01873625 NCT01663116 NCT03171194 NCT01741857	[42] [43] [44] [45] [46] [47] [48]
**Cardiovascular** **disease**	Type 2 Diabetes Myocardial infarction Heart failure Ischemic stroke	Allo UC Auto BM Auto AT Allo BM Auto BM Auto BM Auto BM Auto BM Allo BM Allo BM Allo BM Allo UC Auto BM	Systemic Local Local Systemic Local Local Local Local Syst/Local Local Systemic Systemic Systemic	2 2 N.A 1/2 2 2/3 2/3 1/2 1/2 2 2 1/2 2	**Safe and clinical efficacy** **Safe and clinical efficacy** **Safe and clinical efficacy** **Safe and clinical efficacy** **Safe and clinical efficacy** **Safe and clinical efficacy** **Safe and no efficacy** **Safe and clinical efficacy** **Safe and no efficacy** **Safe and clinical efficacy** **Safe and clinical efficacy** **Safe and clinical efficacy** **Safe and clinical efficacy**	NCT02302599 NCT02384018 NCT03276312 NCT01576328 NCT01759823 NCT01392105 NCT04421274 NCT01076920 NCT00883727 NCT02013674 NCT01436487 NCT01739777 NCT00875654	[49] [50] [51] [52] [53] [54] [55] [56] [57] [58] [59] [60] [61]
**GvHD**	Acute GvHD	Allo BM Allo BM Allo BM	Systemic Systemic Systemic	3 3 1	**Safe and differential efficacy** **Safe and clinical efficacy** **Safe and clinical efficacy**	NCT00366145 NCT02336230 **X**	[62] [63] [64]
**Intestinal bowel** **disease**	Crohn’s disease	Allo BM Allo AT	Local Local	1/2 3	**Safe and clinical efficacy** **Safe and clinical remission**	NCT01144962 NCT01541579	[65] [66]
**Organ** **transplantation**	Kidney transplantation	Auto BM Auto BM	Systemic Systemic	1/2 N/A	**Safe and clinical efficacy** **Safe and clinical efficacy**	NCT00734396 NCT00658073	[67] [68]
**Neuro-degenerative disease**	Amyotrophic lateral sclerosis Multiple sclerosis	Auto BM Auto BM Auto BM Auto BM Allo UC Auto NP Auto BM Auto BM Auto BM Auto BM	Systemic Syst/Local Syst/Local Systemic Systemic Local Syst/Local Systemic Systemic Syst/Local	1/2 1 1/2 1 1/2 1 1/2 1/2 2 2	**Safe and clinical efficacy** **Safe and clinical efficacy** **Safe and clinical efficacy** **Safe and unknown efficacy** **Safe and clinical efficacy** **Safe and clinical efficacy** **Safe and clinical efficacy** **Safe and clinical efficacy** **Safe and trend for efficacy** **Safe and clinical efficacy**	NCT04821479 NCT01759797 NCT04823000 NCT00813969 NCT02034188 NCT01933802 NCT00781872 NCT01745783 NCT10228266 NCT02166021	[69] [70] [71] [72] [73] [74] [75] [76] [77] [78]
**Viral infection**	SARS-CoV-2	Allo AT Allo **X** Allo BM	Systemic Systemic Systemic	1 1 1/2	**Safe and clinical efficacy** **Safe and no efficacy** **Safe and clinical efficacy**	NCT04276987 NCT04535856 NCT05019287	[79] [80] [81]

Abbreviations: bone marrow (BM); adipose tissue (AT); umbilical cord (UC); NP, neural progen-itors (NP); allogeneic (allo); autologous (auto); not available (NA).

## Data Availability

Not applicable.
